# *Cortinarius* barcoding database of Western Siberia and adjacent areas

**DOI:** 10.3897/BDJ.14.e196734

**Published:** 2026-07-07

**Authors:** Nina Filippova, Tatiana M. Bulyonkova, Elena Zvyagina, Dmitry Ageev, Elena Rudykina, Alexandra Mingalimova

**Affiliations:** 1 Yugra State University, Khanty-Mansiysk, Russia Yugra State University Khanty-Mansiysk Russia; 2 A.P. Ershov Institute of Informatics Systems Russian Academy of Sciences, Novosibirsk, Russia A.P. Ershov Institute of Informatics Systems Russian Academy of Sciences Novosibirsk Russia; 3 Moskow State University, Moskow, Russia Moskow State University Moskow Russia; 4 Independent researcher, Novosibirsk, Russia Independent researcher Novosibirsk Russia

**Keywords:** DNA, ectomycorrhizal fungi, ITS, molecular identification, peatlands, phylogenetics, pine forests, Russia, taxonomy, voucher specimens, sanger sequencing, webcaps

## Abstract

**Background:**

The genus *Cortinarius* (Pers.) Grays. is a highly diverse and ecologically crucial group of ectomycorrhizal fungi in boreal forests. Despite a long history of mycological study in Russia, a comprehensive, molecularly validated inventory of its diversity in Western Siberia has been lacking. Global genetic resources are essential for modern fungal research, yet such a curated, regional dataset for this complex genus has not been previously available for this region.

**New information:**

This paper describes a curated database of 624 *Cortinarius* specimens from Western Siberia and adjacent regions, resulting in 624 high-quality ITS sequences. The dataset includes detailed collection metadata, morphological descriptions and photographic documentation, all standardised and linked to DNA sequence data originally managed in Specify 7. The sequences were processed through a rigorous bioinformatics pipeline with strict quality controls and assigned provisional taxonomy using a defined BLAST protocol against international reference databases. The complete dataset, including raw sequences, specimen data and collection images, has been deposited in international repositories (Global Biodiversity Information Facility (GBIF) (https://doi.org/10.15468/4v8km8), Sequence Reads Archive (SRA) and GenBank), providing a foundational resource for future taxonomic, phylogenetic and ecological studies on this key fungal genus in Western Siberia.

## Introduction

The genus *Cortinarius* s.l. (commonly known as webcaps) is one of the largest and most complex genera of agaricoid basidiomycetes, with over 3000 described species and the true diversity estimated to reach up to 5000 species (Gallone et al. 2024). Members of this genus play a crucial role in many forest ecosystems by forming mycorrhizal associations with various tree species and are the most species-rich group of ectomycorrhizal fungi in boreal and temperate forests (Bödeker et al. 2014, Clemmensen et al. 2014, Lindahl et al. 2026).

Due to their high species diversity and intricate taxonomy, *Cortinarius* is of significant interest in mycological research. In recent decades, the application of molecular genetic methods has allowed for the revision of classification within many groups of the genus, revealing cryptic species and clarifying phylogenetic relationships (Garnica et al. 2017, Soop et al. 2019, to mention a few). A major phylogenomic study by Liimatainen et al. (2020) proposed splitting *Cortinarius* into ten genera, but this classification was later challenged due to unresolved backbone branches and extensive gene-tree/species-tree conflicts (Gallone et al. 2024). Consequently, most researchers and taxonomic databases (e.g. IndexFungorum, Catalogue of Life, GBIF) continue to recognise *Cortinarius* in the broad sense, which is the approach we follow in this paper.

The study of webcaps in Russia started about a century ago (see Nezdoiminogo (1996) for a historical review). Although studies on this genus in Western Siberia date back to the early 20^th^ century, a comprehensive or at least representative checklist supported by modern molecular data has not been compiled. According to a major revision of published occurrences of agaricoid and boletoid fungi of Russia, there are currently around 414 species of *Cortinarius* recorded in total for Russia and about 280 of them in Siberia (Bolshakov et al. 2021).

Modern mycological research requires integration of biodiversity data from various sources. Global information systems, such as GenBank, GBIF, UNITE (Abarenkov et al. 2023) and ENA (European Nucleotide Archive), play a key role in storing and analysing molecular and morphological data about fungi. Standardisation of rich data presentation (including collection metadata, molecular genetic data, geographic coordinates and photographs of macro- and micromorphological features) enables large-scale phylogenetic and ecological studies and clarifies taxonomic uncertainty (Abarenkov et al. 2022).

**Figure 1.**
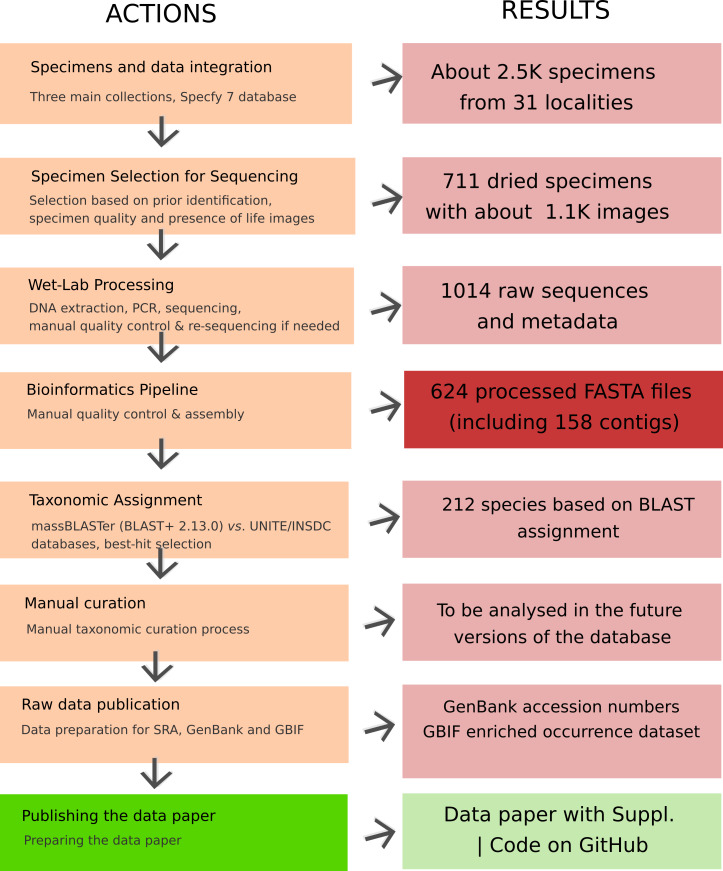
Actions and results of preparing data on the *Cortinarius* barcoding database of West Siberia.

The aim of this study is to present a new *Cortinarius* specimen and sequence database, which includes samples from Russia (particularly Western Siberia) and to outline the methods used for their collection, processing and deposition in international databases (Fig. 1).

## Sampling methods

### Step description

The samples for sequencing were obtained from several personal mycological collections in Western Siberia. All specimens were deposited in the Biological Collection of Yugra State University (YSU), where they underwent cataloguing and subsequent molecular-genetic processing. The collection comprises approximately 2500 specimens of *Cortinarius* s.l., with about 700 selected for sequencing. The work, involving collection consolidation, DNA extraction and sequence generation, spanned three years.

**Specimen collection preparation**

**Figure 2.**
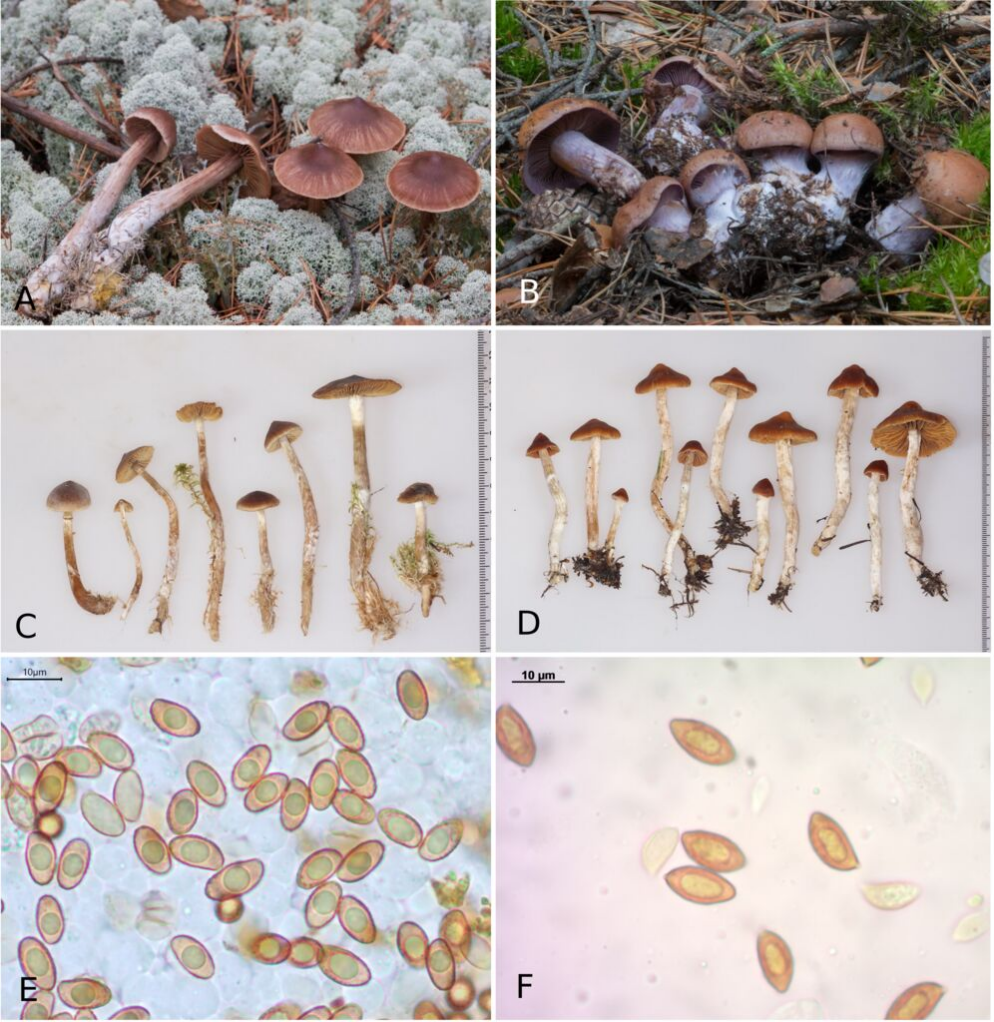
Examples of different image types uploaded to the database: field photographs of specimens, macro-morphological features and spore microphotographs. **A** *C. glandicolor* YSU-F-12978; **B** *C. epipurrus* YSU-F-13110; **C** *C. kauffmanianus* YSU-F-05991; **D** *C. mammillatus* YSU-F-06151; **E** *C. cinnanomeus* YSU-F-12166; **F** *C. collinitus* YSU-F-05770.

Fresh material was collected and characterised according to the standard methods used in fungal taxonomy (Clémençon et al. 2004). Fresh collections were photographed and described in the field. Morphological descriptions of fruiting bodies were based on the examination of fresh and dried material. A standard set of reagents (5% KOH, 1% Congo Red solution and Melzer’s reagent) and a Zeiss AxioStar microscope with a digital camera AxioCam ERc5s and Zeiss AxioVision 4.8.2 software were used for the study and documentation of microstructures. The specimen data were stored in the Specify 7 collection database, organised into dedicated tables for samples, collection events, personnel, DNA extracts, associated images (Fig. 2), morphological descriptions and related measurement files.

**DNA аmplification and sequencing**

The PCR was made using the TransDirect® Plant Tissue PCR Kit without DNA purification. PCR reactions were performed in 20 μl of reaction mixtures containing 4 μl of ScreenMix (Evrogen), 0.2 μl of each PCR primer, 14 μl of deionised H2O and 1.6 μl of template DNA. For amplification of the ITS region, the primers ITS1F (Gardes and Bruns 2008) and ITS4 (White et al. 1990) were used. PCR cycle parameters were as follows: initial denaturation for 5 min at 95°C, 30 cycles (denaturation for 20 sec at 95°C, primers annealing for 30 sec at 54°C, extend DNA for 60 sec at 72°C) and final extension for 7 min at 72°C. PCR and sequence reaction products were purified using ExSPure (SkyGen), CleanMag DNA (Evrogen) and Dynabeads™ Sequencing Clean Up kits. Sequencing was performed with the BrilliantDye™ Terminator (v.3.1) Cycle Sequencing Kit (NimaGen) using the Applied Biosystems® Sanger Sequencing 3500 Series Genetic Analyzer. The obtained sequences underwent manual quality assessment. For sequences failing quality thresholds, re-sequencing was performed (either in the forward or reverse direction, as needed).

**Bioinformatics sequence processing methods**

Raw sequences were processed with strict quality thresholds (minimum length: 50 bases, average quality: Q ≥ 20, end quality: ≥ 15). A sliding window approach (5 bp) was used to determine reliable sequence boundaries. Sequences were trimmed at positions where average quality dropped below threshold values. Forward and reverse sequences were aligned using the Needleman-Wunsch algorithm (parameters: match = 2, mismatch = -3, gap_open = -5, gap_extend = -2). Contigs were assembled with ≥ 20 bp overlap, with conflicting positions resolved by prioritising forward-read nucleotides. For single-read samples (forward or reverse only), truncated sequences were retained. All sequences that failed automated quality control were inspected and corrected manually where possible. For each sequence, the following metrics were computed to estimate the resulting sequence quality: length and count of ambiguous bases (N), percentage of N bases and composite quality score: *(length × 0.4) - (%N × 0.6)*, classified as failed, low, medium, good or excellent.

The taxonomic assignment of sequences was performed through analysis with massBLASTer (BLAST+ 2.13.0) on the PlutoF platform using the UNITE (fungi) and INSDC (fungi) databases with the 'Similar sequences (fast)' algorithm parameters. The exported BLAST results were incorporated into the original sequence table for further analysis. Several new fields were created in the dataset: a 'Taxon' field for recording the best taxonomic match (best hit), an 'other taxa' field containing all remaining species-level taxonomic assignments from the BLAST results for each sequence and fields for quantitative BLAST parameters, including percentage similarity and other relevant metrics.

The selection of best hits followed strict criteria. Only matches identified to the species level with proper binomial nomenclature were considered, while entries containing markers like sp., cf. or aff. were excluded from consideration. The primary selection priority was given to type specimens (is_type = True) using the NCBI information system of type specimens. When multiple species-level matches existed, the secondary selection criterion was the highest percentage identity amongst the qualifying matches. This approach ensured the most reliable taxonomic assignments while maintaining traceability of all potential matches and their respective alignment quality metrics. The complete set of BLAST parameters used in this analysis is documented for reproducibility purposes (Filippova 2025b).

The resulting annotated table contains several key components: the original sequence data, filtered best hits meeting the established criteria, all alternative species-level matches and comprehensive alignment statistics (Suppl. material 1). This structure facilitates both the immediate taxonomic interpretation and potential future re-evaluation of sequence assignments. The methodology emphasises scientific rigour through its transparent selection hierarchy and preservation of all relevant matching data. Quantitative metrics from the BLAST alignments remain available for assessing match confidence and performing subsequent quality control analyses.

For subsequent submission to GenBank, SRA and GBIF databases, we employed taxon names, based on automated classification complemented by percentage identity in identificationRemarks. Alternatively, sequences showing 76-97% similarity to reference entries could be assigned to the genus level only. Low-percentage identity flags potentially novel taxa that may require further phylogenetic investigation and formal description through focused studies on specific taxonomic groups.

Analysis code is available on GitHub (Filippova 2025a) and Zenodo (Filippova 2025b).

**Data Storage**

Specimen data with images and associated processed sequences were deposited as an enriched occurrence dataset in GBIF. Raw sequence files in fastq.gz format (with sample metadata) were submitted to the SRA portal. Processed sequences were also uploaded to GenBank. The data analysis code is made available on GitHub (Filippova 2025a).

## Geographic coverage

### Description

**Figure 3.**
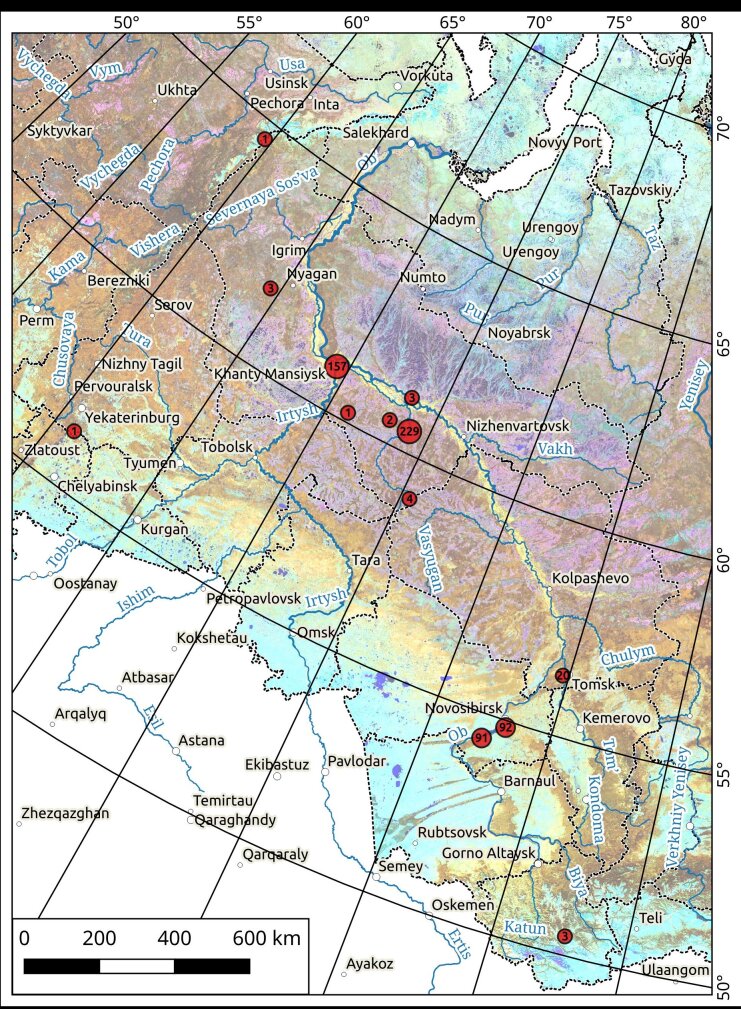
Distribution map of sequenced specimens occurrences in *Cortinarius* barcoding database on MODIS satellite image after clusterisation to 5 km radius.

The sequenced specimens were collected primarily in the Khanty-Mansi Autonomous Okrug (63%) and Novosibirsk Oblast (32%), with a small number from four other regions of Western Siberia. The samples were obtained from 31 localities (standardised to a 5 km radius), with 85% concentrated in just six key sites: the vicinity of Ugut Village (22%), Novosibirsk City (16%), Shapsha Village (15%), Mukhrino research station (12%), Karakansky Bor (12%) and Yugansky Nature Reserve (9%) (Fig. 3).

### Coordinates

44.0070 and 66.5532 Latitude; 33.0886 and 145.7013 Longitude.

## Usage licence

### Usage licence

Other

### IP rights notes

Creative Commons Attribution (CC-BY 4.0) License

## Data resources

### Data package title

*Cortinarius* barcoding database of Western Siberia and adjacent areas

### Resource link

https://doi.org/10.15468/4v8km8

### Alternative identifiers

https://www.gbif.org/dataset/e1c0913a-49b2-4d39-8dde-d840e93ffbb3

### Number of data sets

2

### Data set 1.

#### Data set name

*Cortinarius* barcoding database of Western Siberia and adjacent areas

#### Data format

Darwin Core

#### Character set

UTF-8

#### Download URL

http://ipt.ugrasu.ru:8080/resource?r=cortdb&v=1.24

#### Description

The dataset (Filippova et al. 2025) presents a comprehensive DNA barcode sequence collection for the fungal genus *Cortinarius* (webcaps) from Western Siberia and adjacent regions of Russia. The dataset integrates collection metadata (geography, habitat, collectors), molecular data (raw and processed sequences) and morphological data (photographs, descriptions).

**Data set 1.** 

Column label	Column description
occurrenceID	A unique identifier for the occurrence.
basisOfRecord	The nature of the record (preserved specimen).
collectionCode	The collection code for voucher specimens (five collection codes listed in the dataset).
associatedSequences	Accession number of the sequence deposited in NCBI.
catalogNumber	The collection catalogue number.
otherCatalogNumbers	Alternative catalogue numbers of duplicate specimens.
scientificName	The full scientific name.
identifiedBy	A person who last identified the specimen.
identificationRemarks	Identification based on BLAST analysis: showing the nearest sequence (percentage identity) and other closely related taxa. (for example: ID based on BLAST nearest sequence GU363496 (91.90%) (specimen), other nearest taxa: *Calonarius aureofulvus* | *Calonarius cupreorufus* | *Calonarius flavobulbus* | *Calonarius saxamontanus*).
dateIdentified	The date on which the last identification had been made.
country	The name of the country (Russia).
countryCode	The standard code for the country (RU).
stateProvince	The name of administrative region of second order (Region).
county	The name of administrative region of third order (Region).
locality	The locality name or topographic description, in English.
decimalLatitude	The geographic latitude.
decimalLongitude	The geographic longitude.
geodeticDatum	The geodetic datum of the coordinate.
eventDate	The date during which a collection occurred.
habitat	The habitat where the specimen was found.
recordedBy	The person who recorded the occurrence.
eventRemarks	The substrate or mycorrhizal partner on which the specimen was collected.
associatedMedia	Photographs of the specimen in live form and/or micrographs.
kingdom	The taxon kingdom (fungi).
taxonRank	The taxonomic rank of the ScientificName.
coordinateUncertaintyInMetres	The uncertainty of the decimalLatitude and decimalLongitude, in metres.
occurrenceID (dna-derived table)	A unique identifier for the occurrence.
url (dna-derived table)	Accession number of the sequence deposited in NCBI.
target_gene (dna-derived table)	Target marker (ITS region).
DNA_sequence (dna-derived table)	The DNA sequence.
pcr_primer_name_forward (dna-derived table)	Name of the forward PCR primer.
pcr_primer_forward (dna-derived table)	Forward PCR primer that was used to amplify the sequence.
pcr_primer_name_reverse (dna-derived table)	Name of the reverse PCR primer.
pcr_primer_reverse (dna-derived table)	Reference for the primers.
seq_meth (dna-derived table)	Sequencing method/platform used (Sanger).
pcr_primer_reference (dna-derived table)	Reference for the primers.

### Data set 2.

#### Data set name

The Supplementary 1 (best hit results with analytical parameters) fields description

#### Data format

tsv table

#### Character set

UTF-8

#### Description

The resulting annotated table contains several key components: the original sequence data, filtered best hits meeting the established criteria, all alternative species-level matches and comprehensive alignment statistics.

**Data set 2.** 

Column label	Column description
catNumber	Catalogue number of specimen.
collectionCode	Collection code.
otherCatNumbers	Catalogue numbers of dublicate specimens in other collections.
seqResult	Sequencing result based on PCR results and sequence quality assessment (PCR failed, seq failed).
traceFileName	Original file name of sequence.
region	Target region (ITS).
direction	Target region direction (F, R).
nameManual	Original taxon name, based on manual BLAST or phylogenetic analysis.
seqFasta	Original sequence based, on manual sequence processing.
length	Original sequence length.
fastaLength	Processed fasta sequence length.
mean_quality	Original sequence quality.
result_type	Result of assembled contigs or used single reads for automatic sequence processing (contig, F_only, R_only).
auto_sequence	Automatic sequence, processed by pipeline.
identity_percent	Comparison result of manual and automatic sequence.
seq_length	Final sequence length.
n_count	Number of ambiguous bases (N) in final sequence.
n_percentage	Percentage of ambiguous bases (N) in final sequence.
quality_score	Calculated quality score.
quality_category	Quality score class (Low, Medium, Good, Excellent).
Reference	The nearest (best hit) BLAST search reference number.
SH:0.5%	Species Hypothesis threshold at 0.5% distance in UNITE system.
SH:1.0%	SH at 1.0%.
SH:1.5%	SH at 1.5%.
taxonName	Best hit (based on BLASTN) taxon name.
Score	The alignment score indicating the quality of the match between query and reference sequences.
MisM	The number of mismatches in BLAST query.
Prcnt	Percentage identity - the proportion of identical bases in the aligned region between query and reference sequences (0-100%).
is_type	Type specimen of the nearest reference number.
All_Taxa	All other binomial taxa from BLAST search results.
identificationRemarks	Combined best hit Reference, Prcn (is_type) and all BLAST search species (example: "ID based on BLAST nearest sequence OQ366595 (99.36 %) (specimen), other nearest taxa: *Cortinarius malachius* | *Cortinarius pseudobiformis* | *Cortinarius quarciticus*").
identification_match	BLASTn and original taxon name match.

## Additional information

### Specimen analysis

The sequenced specimens were collected primarily in the Khanty-Mansi Autonomous Okrug (63%) and Novosibirsk Oblast (32%), with a small number from four other regions of Western Siberia and other territories. The samples were obtained from 31 localities (standardised to a 5 km radius), with 85% concentrated in just six key sites: the vicinity of Ugut Village (22%), Novosibirsk City (16%), Shapsha Village (15%), Mukhrino research station (12%), Karakansky Bor (12%) and Yugansky Nature Reserve (9%) (Fig. 3).

The collection spans specimens gathered between 2005 and 2024. The main collectors were T.M. Bulyonkova (54%), N.V. Filippova (23%) and E.A. Zvyagina (16%), with ten other collectors contributing smaller number of samples.

The specimens represent three dominant ecosystem types: dark coniferous forests (boreal taiga forests with the arboreal layer dominated by *Picea obovata* Ledeb., *Pinus sibirica* Du Tour and *Abies sibirica* Ledeb., typically with an admixture of *Betula pubescens* Ehrh., *Pinus sylvestris* L. and *Populus tremula* L.), raised *Sphagnum* bogs (dominated by dwarf forms of *P. sylvestris*) and a variety of almost pure Scots pine (*P. sylvestris*) forests on poor, acidic, as well as calcareous sandy soils (locally known as “bors”).

### Sequence analysis

**Figure 4.**
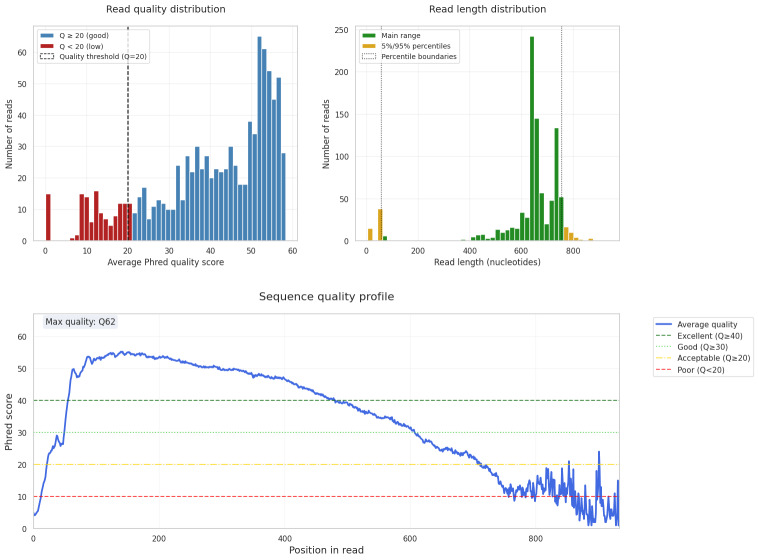
Sequence quality and length analysis before processing. **(A) Left panel**: Read quality distribution. Histogram displays the distribution of mean Phred quality scores across all sequencing reads. Sequences with acceptable quality (Q ≥ 20) are shown in blue, while those below the quality threshold (Q < 20) are highlighted in red; **(B) Right panel**: Read length distribution. Histogram shows the nucleotide length distribution of all sequences, with the main distribution (between 5^th^ and 95^th^ percentiles) displayed in green; **(C) Bottom panel**: Position-specific quality profile. Line plot demonstrates how average base call quality (Phred score) changes across read positions. Quality thresholds are marked with horizontal lines: excellent (Q ≥ 40, dark green), good (Q ≥ 30, lime green), acceptable (Q ≥ 20, gold) and poor (Q < 20, red).

A total of 711 specimens were processed for sequencing, yielding 1014 raw sequences. PCR amplification (performed twice for problematic cases) failed for only 44 specimens (6% of all sequenced samples). Sequencing was eventually unsuccessful for 149 specimens (10% of total), including five cases of contamination and 144 poor-quality sequences (mean Q < 20, discarded after quality control) (Fig. 4).

**Figure 5.**
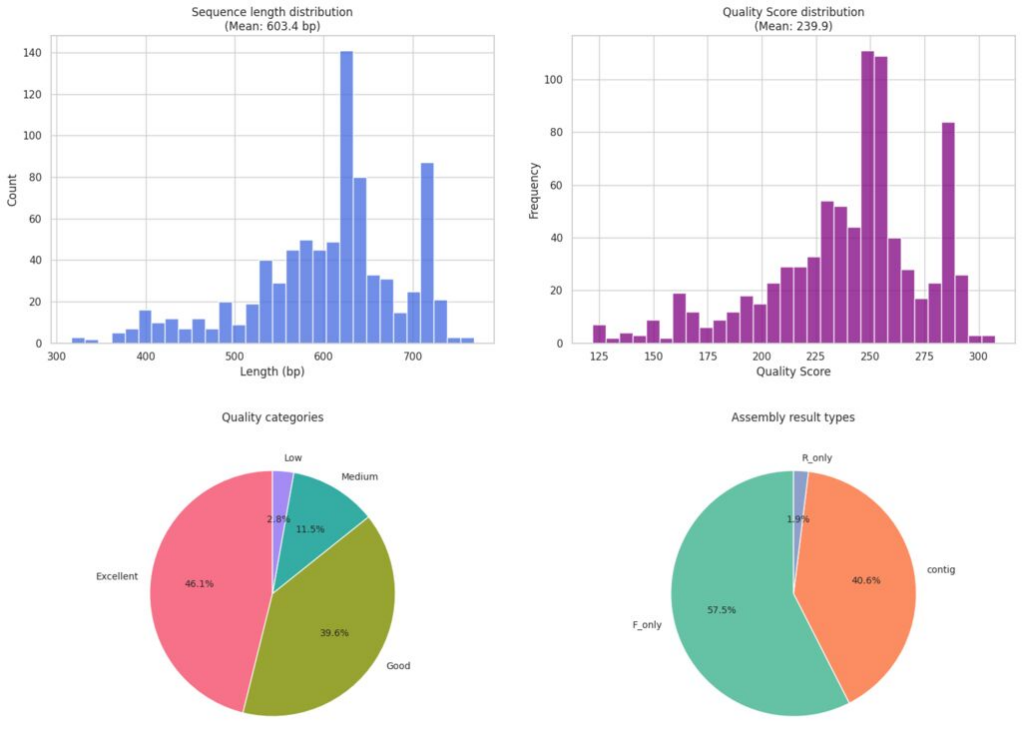
Quality metrics of processed sequences based on the composite quality score. **A) Top-left**: Length distribution histogram showing processed sequence lengths in base pairs (bp); **(B) Top-right**: Distribution of composite quality scores (calculated as (length × 0.4) – (%N × 0.6)); **(C) Bottom-left**: Composite quality category composition (failed, low, medium, good, excellent), based on the composite score thresholds; **(D) Bottom-right**: Assembly result type breakdown.

Processing of 821 raw sequences resulted in 624 processed fasta sequences, with a mean sequence length of 622 nucleotid bases (Fig. 5). The composite quality score (calculated as (length × 0.4) – (%N × 0.6)) averaged 44, with 81% of sequences classified as high-quality (Excellent: 382; Good: 328) and only 3% (24 sequences) falling into low-quality categories. Assembly success metrics showed 159 contigs (40% of total) with both forward and reverse reads aligned, while 453 sequences (58%) contained only forward reads and 12 (2%) only reverse reads.

### Taxonomic analysis

The taxonomic coverage of the database includes 213 species of *Cortinarius* s.l. Of the identified taxa, approximately 17% (106 specimens) show high sequence affinity to the ITS regions of type specimens. The species representation varies significantly: 94 species are represented by only one sequenced specimen, 43 species by two specimens, 69 species by 3–9 specimens and eight species by 10–21 sequenced specimens.

### Conclusions and future goals

The database consolidates processed sequence data (ITS) to support research in diversity, ecology and systematics of *Cortinarius* s.l. in Western Siberia. While many of the provisional species identifications represent novel regional occurrences, further in-depth phylogenetic validation is required to assess poorly-resolved and cryptic species complexes. While not covering the full taxonomic diversity of the genus in the region, the database serves two complementary purposes: (1) as a well-curated collection of sequenced specimens with detailed metadata, contributing to knowledge of *Cortinarius* diversity in Western Siberia; and (2) as a regional DNA barcode sequence collection that can be used in bioinformatic pipelines for species identification and community analyses, particularly for studies focusing on this geographic region.

The database will be further developed in the following key directions:

1. Expanding taxonomic coverage by incorporating additional collections and species;
2. Investigating specimens with low similarity to existing references (potential novel taxa);
3. Expanding geographical coverage by including specimens from other regions.

## Supplementary Material

132E8CE6-AFED-52D9-A74E-9C97BBB8D4FA 10.3897/BDJ.14.e196734.suppl1 Supplementary material 1 Best hit results with analytical parameters Data type tsv table Brief description The resulting annotated table contains several key components: the original sequence data, filtered best hits meeting the established criteria, all alternative species-level matches and comprehensive alignment statistics. File: oo_1573807.tsv https://binary.pensoft.net/file/1573807 Filippova, NV

## References

[B13603410] Abarenkov Kessy, Kristiansson Erik, Ryberg Martin, Nogal-Prata Sandra, Gómez-Martínez Daniela, Stüer-Patowsky Katrin, Jansson Tobias, Põlme Sergei, Ghobad-Nejhad Masoomeh, Corcoll Natàlia, Scharn Ruud, Sánchez-García Marisol, Khomich Maryia, Wurzbacher Christian, Nilsson R. Henrik (2022). The curse of the uncultured fungus. MycoKeys.

[B13603430] Abarenkov Kessy, Nilsson R Henrik, Larsson Karl-Henrik, Taylor Andy F S, May Tom W, Frøslev Tobias Guldberg, Pawlowska Julia, Lindahl Björn, Põldmaa Kadri, Truong Camille, Vu Duong, Hosoya Tsuyoshi, Niskanen Tuula, Piirmann Timo, Ivanov Filipp, Zirk Allan, Peterson Marko, Cheeke Tanya E, Ishigami Yui, Jansson Arnold Tobias, Jeppesen Thomas Stjernegaard, Kristiansson Erik, Mikryukov Vladimir, Miller Joseph T, Oono Ryoko, Ossandon Francisco J, Paupério Joana, Saar Irja, Schigel Dmitry, Suija Ave, Tedersoo Leho, Kõljalg Urmas (2023). The UNITE database for molecular identification and taxonomic communication of fungi and other eukaryotes: sequences, taxa and classifications reconsidered. Nucleic Acids Research.

[B14256364] Bödeker Inga T. M., Clemmensen Karina E., de Boer Wietse, Martin Francis, Olson Åke, Lindahl Björn D. (2014). Ectomycorrhizal *Cortinarius* species participate in enzymatic oxidation of humus in northern forest ecosystems. New Phytologist.

[B13588645] Bolshakov Sergey, Kalinina Lyudmila, Palomozhnykh Ekaterina, Potapov Kim, Ageyev Dmitry, Arslanov Salavat, Filippova Nina, Palamarchuk Marina, Tomchin Dmitry, Voronina Elena (2021). Agaricoid and boletoid fungi of Russia: the modern country-scale checklist of scientific names based on literature data. Biological Communications.

[B13588637] Clémençon H, Emmett V, Emmett E (2004). Cytology and plectology of the Hymenomycetes.

[B14256375] Clemmensen Karina E., Finlay Roger D., Dahlberg Anders, Stenlid Jan, Wardle David A., Lindahl Björn D. (2014). Carbon sequestration is related to mycorrhizal fungal community shifts during long‐term succession in boreal forests. New Phytologist.

[B13435127] Filippova NV (2025). *Cortinarius* barcoding database of Western Siberia and adjacent areas – code for data analysis. https://github.com/ninacourlee/cortbarcodingdatabase.git.

[B13696725] Filippova NV (2025). Analysis code and source data for the article (Filippova NV, Bulyonkova TM, Zvyagina EA, Ageev DV, Rudykina EA «*Cortinarius* barcoding database of Western Siberia and adjacent areas»)..

[B13619398] Filippova N, Zvyagina E, Bulyonkova T, Rudykina E, Ageev D (2025). *Cortinarius* barcoding database of Western Siberia and adjacent areas. Occurrence dataset.

[B14256152] Gallone Brigida, Kuyper Thomas W., Nuytinck Jorinde (2024). The genus Cortinarius should not (yet) be split. IMA Fungus.

[B13750870] Gardes M., Bruns T. D. (2008). ITS primers with enhanced specificity for basidiomycetes ‐ application to the identification of mycorrhizae and rusts. Molecular Ecology.

[B13603351] Garnica S., Weiß M., Oertel B., Oberwinkler F. (2017). Phylogenetic relationships of European *Phlegmacium* species (Cortinarius, Agaricales). Mycologia.

[B13603338] Liimatainen Kare, Niskanen Tuula, Dima Bálint, Ammirati Joseph F., Kirk Paul M., Kytövuori Ilkka (2020). Mission impossible completed: unlocking the nomenclature of the largest and most complicated subgenus of *Cortinarius*, Telamonia. Fungal Diversity.

[B14256355] Lindahl Björn D., Clemmensen Karina E., Stendahl Johan, Dahlberg Anders (2026). Long‐term effects of clear‐cutting forestry on ectomycorrhizal fungi in boreal forest. New Phytologist.

[B13603370] Nezdoiminogo EL (1996). Определитель грибов Россиb. Порядок Агариковые. Выпуск 1. Семейство Паутинниковые..

[B13603360] Soop K., Dima B., Cooper J. A., Park D., Oertel B. (2019). A phylogenetic approach to a global supraspecific taxonomy of *Cortinarius* (Agaricales) with an emphasis on the southern mycota. Persoonia - Molecular Phylogeny and Evolution of Fungi.

[B13750861] White T. J., Bruns T., Lee S., Taylor J. (1990). Amplification and Direct Sequencing of Fungal Ribosomal RNA Genes for Phylogenetics. PCR Protocols.

